# *FCRL3* genetic variants drive autoimmune pathogenesis in multiple sclerosis and neuromyelitis optica spectrum disorders

**DOI:** 10.3389/fneur.2025.1552149

**Published:** 2025-07-04

**Authors:** Hui-Fen Huang, Qi-Bing Liu, Yong-Feng Xu, Gui-Xian Zhao, Hai-Peng Liu, Hao Yu, Zhi-Ying Wu

**Affiliations:** ^1^Department of Medical Genetics and Center for Rare Diseases, Second Affiliated Hospital, Zhejiang University School of Medicine and Zhejiang Key Laboratory of Rare Diseases for Precision Medicine and Clinical Translation, Hangzhou, China; ^2^Department of Neurology, Lishui Hospital, Zhejiang University School of Medicine, Lishui, China; ^3^Department of Neurology, First Affiliated Hospital, Fujian Medical University, Fuzhou, China; ^4^Department of Neurology, Huashan Hospital, Shanghai Medical College, Fudan University, Shanghai, China; ^5^Research Centre for Intelligent Healthcare, Faculty of Health and Life Sciences, Coventry University, Coventry, United Kingdom

**Keywords:** multiple sclerosis, neuromyelitis optica spectrum disorders, *FCRL3*, variant, association

## Abstract

**Objective:**

This study aims to investigate the association of Fc receptor-like 3 (*FCRL3*) gene variants with multiple sclerosis (MS) and neuromyelitis optica spectrum disorder (NMOSD) in a Chinese population cohort.

**Methods:**

In Stage 1, 154 MS patients, 109 NMOSD patients, and 301 normal controls were recruited, Sequenom MassARRAY technology was used for genotyping single nucleotide polymorphisms (SNPs). Stage 2 involved an independent cohort of 95 MS patients, 139 NMOSD patients, and 226 normal controls. Two *FCRL3* SNPs (rs7528684 and rs11264799) were determined using allele-specific polymerase chain reaction (PCR) with specific primers.

**Results:**

Allele C of rs7528684 emerged as a protective factor for MS. Allele A of rs11264799 exhibited no significant effect on MS or NMOSD. A notable disparity in rs7528684 genotype distribution was observed between oligoclonal band (OCB)-positive and OCB-negative MS patients. Allele C of rs7528684 exhibited an association with OCB-positive MS patients.

**Conclusion:**

The findings suggest that the *FCRL3* variant (rs7528684) is associated with MS rather than NMOSD. *FCRL3* might significantly contribute to OCB synthesis, while the underlying mechanisms warrant further elucidation.

## 1 Introduction

Multiple sclerosis (MS) and neuromyelitis optica spectrum disorder (NMOSD) are autoimmune inflammatory demyelinating conditions affecting the central nervous system (CNS) that primarily afflict young females (1). While the precise pathogenesis of MS and NMOSD remains incompletely elucidated, it is associated with a spectrum of genetic and environmental risk factors (2, 3). NMOSD often affects only the optic nerve and spinal cord, and can involve vision problems in both eyes. In comparison, MS can affect other locations in the CNS but often one eye. Despite these nuances, the similarities between NMOSD and MS in symptom and pathology make the accurate diagnosis difficult (4). Moreover, their clinical manifestations and pathological profiles exhibit considerable diversity across different populations, posing challenges in the accurate diagnosis.

Supplementary examinations, e.g., cerebrospinal fluid (CSF) analysis and imaging play a vital role in definitive diagnosis of NMOSD and MS (5). CSF analysis is instrumental in detecting intrathecal immunoglobulin synthesis, notably through assessing the immunoglobulin G (IgG) index and CSF-specific oligonucleotide bands (OCB), providing pivotal insights into central nervous system inflammation (6). In contrast to MS, NMOSD exhibits a higher prevalence among East Asians and other non-white populations (7, 8). Most NMOSD patients demonstrate the presence of a serum autoantibody specific to the astrocyte water channel protein aquaporin-4 (AQP4) (9, 10). Revised NMOSD criteria classify the condition into AQP4 antibody-positive (AQP4+) and AQP4 antibody-negative (AQP4-) subtypes, with AQP4 + patients showing a higher propensity for relapses compared to AQP4- individuals (11). Despite the unequivocal role of B cells in antibody production, the genetic underpinnings of this antibody production largely remain elusive.

A recent multi-center genome-wide association study (GWAS) identified several novel loci associated with MS, among which the HLA DRB1*1501 allele showed the strongest association (12). Located in the lq21 region, *FCRL3* encodes a Fc receptor-like (FCRL) molecule exhibiting significant structural homology with Fc region immunoglobulin receptors, implicating its role in various autoimmune diseases such as psoriasis, Sjogren’s syndrome, and autoimmune thyroid disorders (13–15). While the G allele of rs3761959 in *FCRL3* was initially identified as a risk factor for MS in the primary GWAS phase, subsequent replication across diverse ethnic groups yielded inconclusive results, underscoring the influence of genetic diversity (12). Previous case–control studies in the Spanish population revealed that the C allele of rs7528684, situated in the promoter region of *FCRL3*, acted as a protective factor against MS (16, 17). Interestingly, rs7528684 has exhibited strong association with rheumatoid arthritis (RA) and anti-cyclic citrullinated peptide (CCP) antibody positivity in the Japanese population (18), hinting at *FCRL3*’s potential involvement in antibody production in autoimmune diseases. A previous study identified the G allele in the −1901A > G polymorphism and the T allele in the -658C > T polymorphism as genetic risk factors for NMO in the Chinese population. Another study found four SNPs in the *FCRL3* gene (FCRL3_3C, 5C, 6A, 8G) that may increase the risk of NMO in the Han Chinese population (19, 20). However, despite these findings, no study to date has investigated the link between *FCRL3* variants and the presence of antibodies in MS and NMOSD. Moreover, the diverse associations of *FCRL3* variants with MS susceptibility in prior studies highlight the complexity and variability within this genetic context (16, 17, 21).

In this study, our objective was to investigate the potential association of *FCRL3* variants with both MS and NMOSD among individuals within the Chinese population. Additionally, we aimed to assess the relationship between *FCRL3* variants and the presence of OCB as well as AQP4 antibody (AQP4-ab).

## 2 Materials and methods

### 2.1 Subjects

Table 1 provides an overview of the general clinical characteristics of the subjects involved in this study. For Stage 1, we recruited 154 MS patients (88 females and 66 males), 109 NMOSD patients (89 females and 20 males), and 301 normal controls (141 females and 160 males) from the First Affiliated Hospital of Fujian Medical University and Huashan Hospital of Fudan University between October 25, 2007, and February 4, 2012. Among these individuals, the positive rate of oligoclonal bands in MS patients was 58.2%, while the positive rate of AQP4 antibody in NMOSD patients was 57.14%.

**Table 1:** Clinical characteristics of participants in each stage.

	Male/female	Age at analysis	Age at onset	Disease duration	Relapsing–remitting course	OCB positive/total	AQP4-ab positive/total	*p* value	*p* value
		(y)	(y)	(y)	*n* (%)	*n* (%)	*n* (%)	Age (Case/HC)	Sex (case/HC)
Stage 1									
M S (*n* = 154)	66/88	39.71 ± 13.17	32.35 ± 12.69	6.80 ± 5.62	147 (95.45%)	71/122 (58.2%)	0/80 (0.00%)	0.845	0.04
NMOSD (*n* = 109)	20/89	43.81 ± 14.49	36.82 ± 14.39	6.91 ± 7.24	102 (93.58%)	10/67 (14.93%)	44/77 (57.14%)	0.029	3.5 × 10^−10^
NC (*n* = 301)	160/141	40.01 ± 18.23	NA	NA	NA	NA	NA	NA	
Stage 2									
MS (*n* = 95)	42/53	37.08 ± 12.57	33.58 ± 12.28	4.61 ± 5.94	75 (78.95%)	23/50 (46.0%)	0/21 (0.00%)	6.8 × 10^−7^	0.24
NMOSD (*n* = 139)	22/117	45.17 ± 14.42	34.83 ± 15.13	4.27 ± 5.96	106 (76.26%)	3/49 (6.12%)	97/121 (80.17%)	3.5 × 10^−23^	1.3 × 10^−5^
NC (*n* = 226)	84/142	29.59 ± 9.67	NA	NA	NA	NA	NA	NA	
Stage1 + 2									
MS (*n* = 249)	108/141	38.71 ± 12.98	32.81 ± 12.58	6.11 ± 5.82	222 (89.16%)	94/172 (54.7%)	0/101 (0.00%)	0.03	0.44
NMOSD (*n* = 248)	42/206	44.57 ± 14.44	39.62 ± 14.96	5.59 ± 6.72	208 (83.87%)	13/116 (11.2%)	141/198 (71.2%)	1.32 × 10^−13^	2.7 × 10^−15^
NC (*n* = 527)	244/283	35.54 ± 16.00	NA	NA	NA	NA	NA	NA	

MS, multiple sclerosis; NMOSD, neuromyelitis optica spectrum disease; NC, normal controls; NA, not applicable.

AQP4-ab, AQP-4-antibody; OCB, oligoclonal bands.

For Stage 2, a separate cohort consisting of 95 MS patients (53 females and 42 males), 139 NMOSD patients (117 females and 22 males), and 226 normal controls (142 females and 84 males) was enrolled from the Second Affiliated Hospital of Zhejiang University School of Medicine and the First Affiliated Hospital of Fujian Medical University between March 6, 2013, and March 31, 2021. In this stage, the positive rate of oligoclonal bands in MS patients was 46.0%, whereas the positive rate of AQP4 antibody in NMOSD patients increased to 80.17%. All participants met the current diagnostic criteria for either MS or NMOSD (5, 11).

Detection of AQP4 antibodies was conducted using an indirect immunofluorescence assay with a Euroimmun (Germany) kit, following the manufacturer’s instructions. Transfected and non-transfected AQP4 cells were incubated with diluted serum and FITC-labeled goat anti-human IgG successively. A positive result was indicated by distinct fluorescence on the surface of AQP4-transfected cells, matching the AQP4 expression pattern, with no fluorescence in non-transfected cells. Oligoclonal band detection was conducted using the isoelectric focusing technique followed by agarose gel electrophoresis. Ethical approval for this study was obtained from the Ethics Committees of the mentioned research centers, and informed consent was obtained from all participants or their guardians.

### 2.2 Two-stage SNP genotyping

Genomic DNA extraction from peripheral blood mononuclear cells was conducted using the QIAamp DNA Blood Minikit (QIAGEN, Hilden, Germany) according to the manufacturer’s instruction. All participants underwent genotyping utilizing the Sequenom MassArray system. Four *FCRL3* variants (rs7528684, rs11264799, rs3761959, and rs7522061), previously associated with MS (12), were selected from the GWAS database.

Stage 1 involved genotyping these four SNPs in 154 MS patients, 109 NMOSD patients, and 301 controls at the Fudan-Van Andel Research Institute (VARI) Center (School of Life Science, Fudan University, China). This was carried out using the matrix-assisted laser desorption/ionization time of flight mass spectrometry (MALDI-TOF MS) platform (MassArray TM, Sequenom Inc., San Diego, CA, USA), following a previously established method (22). PCR and extension primers were designed using MassArray Assay Design 3.1 software (Sequenom, San Diego, CA, USA) (Supplementary Table 1).

Based on Stage 1 results, two SNPs of *FCRL3* were chosen for replication in Stage 2, encompassing 95 MS patients, 139 NMOSD patients, and 226 normal controls. This replication stage employed allele-specific PCR with the designated primers (Supplementary Table 2).

### 2.3 Statistical analysis

The Hardy–Weinberg equilibrium was tested using the Chi-squared test. To evaluate differences in allele and genotype frequencies between patients and controls, the Chi-squared test or Fisher’s exact test was employed. Utilizing a binary logistic regression model adjusted for age and gender, odds ratios (ORs) and their corresponding 95% confidence intervals (CIs) were estimated.

Linkage disequilibrium (LD) was determined using SHEsis software (23) available at http://analysis2.bio-x.cn/SHEsisMain.htm. All statistical analyses were conducted using SPSS 23.0 software. Statistical significance was set at a *p*-value less than 0.05.

## 3 Results

### 3.1 Genotyping of four SNPs in MS, NMOSD and control groups

The four SNPs (rs7528684, rs11264799, rs3761959, and rs7522061) within *FCRL3* demonstrated Hardy–Weinberg equilibrium in both MS/NMOSD cases and controls (*p* > 0.05) (Table 2). Comparing the gene frequency distribution of these four SNPs between the MS group and the control group revealed significant associations: rs7522061 (*p* = 0.0003, OR = 0.593), rs3761959 (*p* = 0.0005, OR = 0.601), rs7528684 (*p* = 0.0007, OR = 0.616), rs11264799 (*p* = 0.189, OR = 0.778). The genomic locations of these selected *FCRL3* variants were as follows: rs7522061 located in exon 3, rs3761959 in intron 2, and rs11264799 along with rs7528684 in the promoter region (−110 position and −169 position, respectively).

**Table 2:** Hardy–Weinberg equilibrium of *FCRL3* SNPs in MS, NMOSD and NC in Stage 1.

SNP	MS (*n* = 154)		NMOSD (*n* = 109)		NC (*n* = 301)	
Genotype/Allele	Actual	Expected	Actual	Expected	Actual	Expected
	*n* (%)	*n* (%)	*n* (%)	*n* (%)	*n* (%)	*n* (%)
rs7522061						
AA	66 (42.9)	62 (40.3)	46 (42.2)	44 (40.4)	82 (27.2)	85 (28.3)
GA	64 (41.5)	71 (46.1)	47 (43.1)	51 (46.8)	155 (51.5)	150 (49.8)
GG	24 (15.6)	21 (13.6)	16 (14.7)	14 (12.8)	64 (21.3)	66 (21.9)
A	196 (63.6)		139 (63.8)		319 (53.0)	
G	112 (36.4)		79 (36.2)		283 (47.0)	
*χ2*	0.688		0.341		0.167	
(*p*)	−0.709		−0.843		−0.92	
rs3761959						
CC	68 (44.1)	63 (40.9)	47 (43.1)	45 (41.3)	86 (28.6)	88 (29.2)
TC	62 (40.3)	71 (46.1)	46 (42.2)	50 (45.9)	153 (50.8)	150 (49.8)
TT	24 (15.6)	20 (13.0)	16 (14.7)	14 (12.8)	62 (20.6)	63 (21.0)
C	198 (64.3)		140 (64.2)		325 (54.0)	
T	110 (35.7)		78 (35.8)		277 (46.0)	
*χ2*	1.163		0.237		0.061	
(*p*)	−0.559		−0.888		−0.97	
rs11264799						
GG	94 (61.0)	93 (60.4)	72 (66.0)	73 (67.0)	162 (53.8)	164 (54.5)
AG	51 (33.1)	53 (34.4)	34 (31.2)	32 (29.4)	120 (39.9)	116 (38.5)
AA	9 (5.9)	8 (5.2)	3 (2.8)	4 (3.6)	19 (6.3)	21 (7.0)
G	239 (77.6)		178 (81.7)		444 (73.8)	
A	69 (22.4)		40 (18.3)		158 (26.2)	
*χ2*	0.103		0.21		0.18	
(*p*)	−0.95		−0.9		−0.914	
rs7528684						
TT	68 (44.1)	63 (40.9)	45 (41.3)	44 (40.4)	87 (28.9)	90 (30.0)
CT	62 (40.3)	71 (46.1)	48 (44.0)	50 (45.9)	154 (51.2)	149 (49.4)
CC	24 (15.6)	20 (13.0)	16 (14.7)	15 (13.7)	60 (19.9)	62 (20.6)
T	198 (64.3)		138 (63.3)		328 (54.5)	
C	110 (35.7)		80 (36.7)		274 (45.5)	
*χ2*	1.163		0.084		0.166	
(*p*)	−0.559		−0.959		−0.92	

Hardy–Weinberg equilibrium in each group was carried out by chi-square analysis (df = 2).

MS, multiple sclerosis; NMOSD, neuromyelitis optica spectrum disorder; NC, normal controls; SNP, single nucleotide polymorphism.

**Figure 1:**
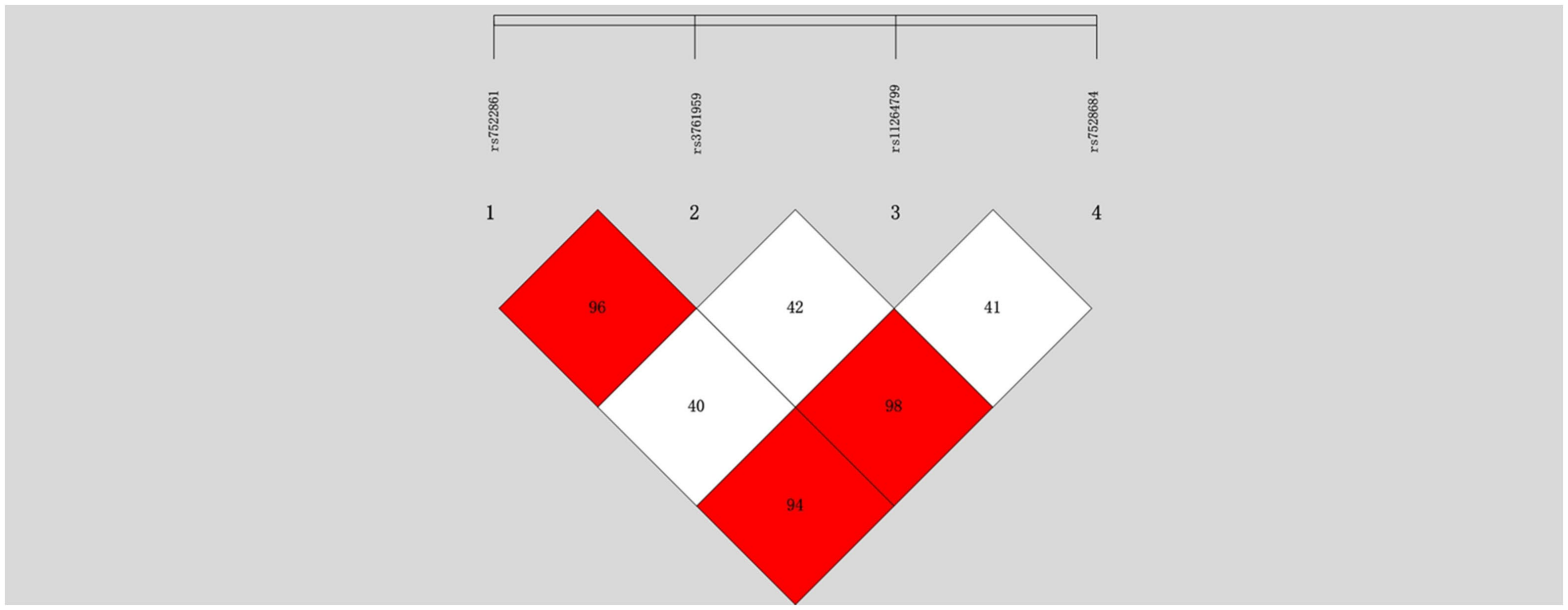
Linkage disequilibrium analysis of four SNPs in the *FCRL3* locus within normal controls.

We observed that rs7528684, positioned in the promoter of *FCRL3*, exhibited high linkage disequilibrium (LD) compared to the other two SNPs (rs7522061 and rs3761959). However, rs11264799, another SNP in the promoter region, displayed relatively lower LD with the aforementioned three SNPs (mean R2 = 0.41) in the controls (Figure 1). Consequently, statistical analyses were focused on rs7528684 and rs11264799.

### 3.2 *FCRL3* is associated with MS in the Chinese population

In Stage 1 (Table 3), the less frequent occurrence of allele C of rs7528684 was noted in both MS and NMOSD patients compared to controls (MS vs. controls: *p* = 0.005, OR = 0.665, 95% CI: 0.501–0.883; NMOSD vs. controls: *p* = 0.024, OR = 0.694, 95% CI: 0.505–0.954). Conversely, allele A of rs11264799 was less common in NMOSD (*p* = 0.020, OR = 0.631, 95% CI: 0.428–0.931) but not in MS when compared to controls. After adjusting for age and gender in a logistic regression model, allele C of rs7528684 demonstrated protective effects for MS in the genotype of dominant model (CT + CC vs. TT, *p* = 0.002, OR = 0.528, 95% CI: 0.352–0.793) but not for NMOSD. Similarly, allele A of rs11264799 acted as a protective factor for NMOSD in the dominant model (AG + AA vs. GG, *p* = 0.005, OR = 0.492, 95% CI: 0.300–0.806) but not for MS.

**Table 3:** Genotype and allele frequencies of rs7528684 and rs11264799 in MS, NMOSD and controls in Stage 1.

SNP/Genotype/Allele	NC	MS	NMOSD	MS versus NC		NMOSD versus NC	
				*p*	OR (95% CI)	*p*	OR (95% CI)
rs7528684							
TT	87 (28.9)	68 (44.1)	45 (41.3)				
CT	154 (51.2)	62 (40.3)	48 (44.0)				
CC	60 (19.9)	24 (15.6)	16 (14.7)				
CT + CC vs. TT	-	-	-	0.002^a^	0.528 (0.352-0.793)	0.058^a^	0.621 (0.380–1.016)
T	328 (54.5)	198 (64.3)	138 (63.3)				
C	274 (45.5)	110 (35.7)	80 (36.7)	0.005^b^	0.665 (0.501–0.883)	0.024^b^	0.694 (0.505–0.954)
rs11264799							
GG	162 (53.8)	94 (61.0)	72 (66.0)				
AG	120 (39.9)	51 (33.1)	34 (31.2)				
AA	19 (6.3)	9 (5.9)	3 (2.8)				
AG + AA vs. GG	-	-	-	0.087^a^	0.711 (0.477-1.061)	0.005^a^	0.492 (0.300–0.806)
G	444 (73.8)	239 (77.6)	178 (81.7)				
A	158 (26.2)	69 (22.4)	40 (18.3)	0.205^b^	0.811 (0.587–1.121)	0.020^b^	0.631 (0.428–0.931)

a Two-sided chi-square test.

b Student’s test.

OR, odds ratio; CI, confidence interval; MS, multiple sclerosis; NMOSD, neuromyelitis optica; NC, normal controls; SNP, single nucleotide polymorphism.

Consequently, rs7528684 and rs11264799 were selected for confirmation in Stage 2. In Stage 2 (Table 4), allele C of rs7528684 remained less prevalent in MS patients compared to controls (*p* = 0.015, OR = 0.647, 95% CI: 0.455–0.920), maintaining its protective association in the dominant model (CT + CC vs. TT, *p* = 0.004, OR = 0.466, 95% CI: 0.276–0.788), consistent with Stage 1 findings. However, no significant association was found between NMOSD and controls. Considering both Stage 1 and Stage 2 collectively (Table 5), the association of rs7528684 strengthened, showing a significant protective association with MS (*p* = 1.93 × 10^−4^, OR = 0.659, 95% CI: 0.529–0.821). Nevertheless, allele A of rs11264799 did not exhibit significant differences in either MS or NMOSD compared to controls (MS vs. controls: *p* = 0.102, OR = 0.808, 95% CI: 0.625–1.044; NMOSD vs. controls: *p* = 0.077, OR = 0.792, 95% CI: 0.612–1.025).

**Table 4:** Genotype and allele frequencies of rs7528684 and rs11264799 in MS, NMOSD and controls in Stage 2.

SNP/Genotype/Allele	NC	MS	NMOSD	MS versus NC		NMOSD versus NC	
				*p*	OR (95% CI)	*p*	OR (95% CI)
rs7528684							
TT	68 (30.1)	44 (46.3)	36 (25.9)				
CT	112 (49.6)	36 (37.9)	77 (55.4)				
CC	46 (20.3)	15 (15.8)	26 (18.7)				
CT + CC vs. TT	-	-	-	0.004^a^	0.466 (0.276-0.788)	0.491^a^	1.229 (0.684–2.208)
T	248 (54.9)	124 (65.3)	149 (53.6)				
C	204 (45.1)	66 (34.7)	129 (46.4)	0.015^b^	0.647 (0.455–0.920)	0.738^b^	1.503 (0.780–1.420)
rs11264799							
GG	133 (58.8)	64 (67.3)	86 (61.9)				
AG	82 (36.3)	26 (27.4)	43 (30.9)				
AA	11 (4.9)	5 (5.3)	10 (7.2)				
AG + AA vs. GG	-	-	-	0.404^a^	0.796 (0.466-1.360)	0.145^a^	0.666 (0.386–1.151)
G	348 (77.0)	154 (81.1)	215 (77.3)				
A	104 (23.0)	36 (18.9)	63 (22.7)	0.255^b^	0.782 (0.512–1.195)	0.914^b^	0.981 (0.687–0.1.400)

a Two-sided chi-square test.

b Student’s test.

OR, odds ratio; CI, confidence interval; MS, multiple sclerosis; NMOSD, neuromyelitis optica; NC, normal controls; SNP, single nucleotide polymorphism.

**Table 5:** Genotype and allele frequencies of rs7528684 and rs11264799 in MS, NMOSD and controls in two Stages.

SNP/Genotype/Allele	Stage 1 + Stage 2						
	NC	MS	NMOSD	MS versus NC		NMOSD versus NC	
				*p*	OR (95% CI)	*p*	OR (95% CI)
rs7528684							
TT	155 (29.4)	112 (45.0)	81 (32.7)				
CT	266 (50.5)	98 (39.4)	125 (50.4)				
CC	106 (20.1)	39 (15.6)	42 (16.9)				
CT + CC vs. TT	-	-	-	0.001^a^	0.489 (0.323–0.740)	0.707^a^	0.914 (0.571–1.463)
T	576 (54.6)	322 (64.7)	287 (57.9)				
C	478 (45.4)	176 (35.3)	209 (42.1)	1.93 × 10^-4b^	0.659 (0.529–0.821)	0.235^b^	0.878 (0.707–1.089)
rs11264799							
GG	295 (56.0)	158 (63.5)	158 (63.7)				
AG	202 (38.3)	77 (30.9)	77 (31.1)				
AA	30 (5.7)	14 (5.6)	13 (5.2)				
AG + AA vs. GG	-	-	-	0.593^a^	0.896 (0.599–1.340)	0.121^a^	0.699 (0.445–1.099)
G	792 (75.1)	393 (78.9)	393 (79.2)				
A	262 (24.9)	105 (21.1)	103 (20.8)	0.102^b^	0.808 (0.625–1.044)	0.077^b^	0.792 (0.612–0.1.025)

^a^Two-sided chi-square test.

^b^Student’s test.

MS, multiple sclerosis; NMOSD, neuromyelitis optica spectrum disease; NC, normal controls; NA, not applicable; OR, odds ratio; CI, confidence interval; OCB, oligoclonal bands.

### 3.3 *FCRL3* is associated with the presence of OCB in MS

Stratification based on the presence of OCB in CSF among MS patients was conducted to further explore the association between immune response and genotype. Table 6 displays a significant disparity in the genotype distribution of rs7528684 between OCB-positive and OCB-negative MS patients (*p* = 0.001). Notably, the C allele of rs7528684 exhibited an association with OCB positivity in MS (*p* = 1.4 × 10^−5^; OR = 0.37; 95% CI: 0.235 to 0.583). Moreover, this allele demonstrated association solely with OCB-positive MS when compared to controls (*p* = 4.1 × 10^–7;^ OR = 0.413; 95% CI: 0.291 to 0.586).

**Table 6:** Genotype and allele frequencies of rs7528684 in all MS and controls in stage 1 and 2 divided by OCB status.

Genotype/Allele	Controls	MS		OCB pos vs. OCB neg			OCB pos vs. Controls			OCB neg vs. Controls		
		OCB pos	OCB neg									
	(*n* = 527) (%)	(*n* = 94) (%)	(*n* = 78) (%)	*p*	OR	95% CI	*p*	OR	95% CI	*p*	OR	95% CI
TT	155 (29.4)	54 (57.5)	24 (30.8)									
CT	266 (50.5)	32 (34.0)	33 (42.3)									
CC	106 (20.1)	8 (8.5)	21 (26.9)	0.001	0.34	0.170–0.646	0.002	0.35	0.177–0.691	0.457	0.532	0.215–1.743
T	576 (54.6)	140 (74.5)	81 (51.9)									
C	478 (45.4)	48 (25.5)	75 (48.1)	1.4 × 10^−5^	0.37	0.235–0.583	4.1 × 10^−7^	0.413	0.291–0.586	0.524	1.116	0.797–1.562

MS, multiple sclerosis; NMOSD, neuromyelitis optica spectrum disease; NC, normal controls; NA, not applicable; OR, odds ratio; CI, confidence interval; OCB, oligoclonal bands.

## 4 Discussion

This investigation delves into the correlation between four *FCRL3* SNPs and MS/NMOSD within the Chinese population. Notably, we observed that genotype and allele frequencies of rs7528684 were protective against MS, specifically highlighting the protective role of the C allele. Confirming our findings, a combined analysis of two distinct cohorts further emphasized this association, aligning with prior research (17). However, divergent findings in other studies have indicated associations of this variant with increased susceptibility to MS (16, 21).

The investigation of OCB positivity in MS, predominantly observed in Caucasian populations (85–98%), contrasted with lower incidences in Asian cohorts (21–63%) (24–28). Our study’s OCB prevalence (54.7%) aligns with previous reports, hinting at potential immunogenetic influences on OCB production. Notably, the association of DRB1*15 with increased OCB frequency across diverse populations, including Japan, Sardinia, Sweden, and Spain, has been reported (29). Our findings further suggest an inverse association between the C allele of rs7528684 in *FCRL3* and the presence of OCB in MS patients. These results propose *FCRL3* as an immunogenetic factor potentially involved in OCB production.

The involvement of FCRL3, primarily expressed in mature B cells within lymph nodes, suggests a potential functional role in B cell maturation regulation. Its cytoplasmic domain, comprising immune receptor tyrosine activating motif (ITAM) and immune receptor tyrosine inhibiting motif (ITIM), hints at its influence on B cell signal transduction by either activating or inhibiting cellular pathways (30). FCRL3 as a functional molecule within the immune system implies its potential role in autoimmune disease pathology, necessitating further investigation to elucidate genotype–phenotype correlations. Notably, the rs7528684 C allele exhibited a strong inverse association with insulinoma-associated antigen 2 (IA2), an anti-islet autoantibody linked to Type 1 diabetes (31). Studies have indicated that the C allele of rs7528684 could enhance nuclear factor-kappa B (NF-κB) binding affinity, subsequently elevating FCRL3 expression (18, 32). Studies reveal lower FCRL3 and interleukin-10 (IL-10) levels in MS patients, yet both are more elevated during remission than in the acute phase. This implies FCRL3 is pivotal for immune protection in MS. It activates the SHP-1 and p38 MAPK pathways, boosting IL-10 secretion and curbing inflammatory factor release (32). FCRL3 expression in B cells is influenced by stimuli such as lipopolysaccharides and CpG, impacting B cell survival, proliferation, and antibody production (33). In MS patients, abnormal B cell activation is linked to oligoclonal band formation. FCRL3 dysfunction may cause excessive B cell activation, driving oligoclonal band formation and disease progression (34).

Regarding rs11264799, the initial Stage 1 analysis suggested the A allele as a protective factor for NMOSD but not for MS, failing confirmation in Stage 2. This outcome contradicts a previous study (19), potentially indicating its role in disease susceptibility when triggered or in interaction with other disease-specific genetic variants or uneven distribution within the population. The observed protective effects of rs7528684 and rs11264799 on MS and NMOSD might be attributed to disease complexity, intricate genetic responses, and disease heterogeneity. These complexities necessitate further exploration to comprehend their precise roles in disease pathogenesis and their potential as therapeutic targets.

This study presents preliminary evidence suggesting that FCRL3 polymorphisms may protect against MS, possibly through neuroprotective mechanisms associated with this genetic location. However, there are several methodological limitations that need to be addressed. First, the small sample size may have restricted the statistical power of our findings, potentially hindering the identification of subtle genetic factors. It is crucial to carry out future replication studies involving larger and more diverse cohorts to confirm these results. Second, as the concept of Myelin Oligodendrocyte Glycoprotein (MOG) antibodies had not been established at the time, we did not conduct further subgroup analyses in AQP4- patients. Third, prospective cohorts with sex-stratified recruitment should be established and subgroup analyses performed to uncover potential gender-specific genetic effects, as the retrospective design revealed an uneven sex distribution within the case group. This raises significant concerns given the well-documented sexual dimorphism in MS epidemiology. Furthermore, due to incomplete clinical information, we were unable to examine the correlation between oligoclonal-IgG bands and clinical data, preventing us from considering all relevant factors that may impact oligoclonal-IgG bands. Regarding disease-modifying therapies (DMT), many patients were not receiving these treatments as the first oral DMT in China was only introduced in 2018 (35). In the future, the correlation between Oligoclonal-IgG bands and clinical information, such as age at onset, gender, years of disease at specimen collection, EDSS, number of recurrences, and the presence or absence of DMT, should be analyzed simultaneously. Such efforts could further clarify how FCRL3-modulated immune pathways contribute to the pathogenesis of MS, thereby enhancing our understanding of this complex disorder.

Exploring the functional role of FCRL3 variants stands as an important and intriguing area further investigation.

## 5 Conclusion

*FCRL3* variants show associations MS and NMOSD within the Chinese population, underscoring the need for future studies to validate these findings across broader ethnic cohorts. Moreover, *FCRL3* might serve as a significant contributor to OCB synthesis, warranting detailed exploration to unravel the precise underlying mechanisms. These findings warrant validation in larger cohorts.

## Data Availability

The datasets presented in this study can be found in online repositories. The names of the repository/repositories and accession number(s) can be found at: https://www.ncbi.nlm.nih.gov/snp/, 90.
