# Supplementary material for: FCRL3 genetic variants drive autoimmune pathogenesis in multiple sclerosis and neuromyelitis optica spectrum disorders
Source: Front Neurol. 2025 Jul 4;16:1552149. doi: 10.3389/fneur.2025.1552149 (PMC12272062; doi:10.3389/fneur.2025.1552149)
Supplement: Supplementary file 1 [file Table_1.docx]

**Supplementary Table 1** PCR and MassEXTEND primers for *FCRL3* SNPs in Stage 1

| SNP | PCR primers | MassEXTEND primers |
| --- | --- | --- |
| rs7522061 | 5′-ACGTTGGATGTTCTCCTTTGAAGGCTGTGG-3′  5′-ACGTTGGATGTTCCCCTGCCTTCATCTTTC-3′ | 5′-TGGACCATGGAGGAT-3′ |
| rs3761959 | 5′-ACGTTGGATGCCTCCGACTTTTTCAGTCTC-3′  5′-ACGTTGGATGCTGCAAGTGTTTCCTTCCTG-3′ | 5′-TTTTTTTCTCCCTACATTACC-3′ |
| rs11264799 | 5′-ACGTTGGATGCAAGGTGTCAAGCATATGCC-3′  5′-ACGTTGGATGTACGGGAAGTCCTTGATCTG-3′ | 5′-CCACAAAGCATATGCCTTTTTGA-3′ |
| rs7528684 | 5′-ACGTTGGATGTTATGAGGCTTCTGAACAGG-3′  5′-ACGTTGGATGGCAGATCTGGGTGAGATTAC-3′ | 5′-ATGAAAATAATACAAATGTACAGATCA-3′ |

**Supplementary Table 2** Allele specific PCR primers for *FCRL3* SNPs in Stage 2

| SNP | Forward primers | Reverse primers |
| --- | --- | --- |
| rs7528684 | 5′- CTGTCTCACCAAAAGCCCGA -3′ | 5′-GGGTGAGATTACGGGAAGTCAT-3′  5′-GGGTGAGATTACGGGAAGTCAC-3′ |
| rs11264799 | 5′-GGTGTCAAGCATATGCCTTTTTGGC-3′  5′-GGTGTCAAGCATATGCCTTTTTGGT-3′ | 5′-GTGCGGGGGATATAAGGGGT-3′ |
